# Scleroderma Renal Crisis in Mixed Connective Tissue Disease With Full Renal Recovery Within 3 Months: A Case Report With Expanding Treatment Modalities to Treat Each Clinical Sign as an Independent Entity

**DOI:** 10.1177/2324709617734012

**Published:** 2017-10-10

**Authors:** Jordana Cheta, Suresh Rijhwani, Harlan Rust

**Affiliations:** 1 Eastern Virginia Medical School, Norfolk, VA, USA

**Keywords:** mixed connective tissue disease, scleroderma renal crisis, thrombotic microangiopathy, renal failure

## Abstract

Mixed connective tissue disease (MCTD) is a rheumatologic overlap syndrome that can present with symptoms of systemic lupus erythematous, scleroderma, and polymyositis. A severe but rare complication that can occur in MCTD is scleroderma renal crisis. With multiple poor prognostic indicators, the renal outcome is usually poor. The clinical and histological picture is one of a thrombotic microangiopathy. Clinical suspicion has to be high for additional thrombotic or autoimmune processes coexisting due to associated morbidity. In this article, we report a rare case of scleroderma renal crisis in a patient with MCTD who we treated with plasma exchange for clinical suspicion for an underlying thrombotic thrombocytopenia and mycophenolate mofetil for MCTD. The patient had multiple poor prognostic indicators yet made a full renal recovery in less than 3 months.

## Introduction

Mixed connective tissue disease (MCTD) is an uncommon rheumatological overlap syndrome with features of systemic lupus erythematous, scleroderma (SSc), and polymyositis. MCTD is distinguished from other overlap syndromes in that it has positive anti-RNP antibodies.^1^ The current standard of care recommends corticosteroids to control signs and symptoms of MCTD, as there is no cure.^2^ Major organ involvement may require high doses of corticosteroids and cytotoxic agents such as cyclophosphamide.^2^ Medications are prescribed to achieve presenting symptom control.

Scleroderma renal crisis (SRC) is an infrequent (seen in 5% to 10% of patients with SSc) but severe complication that rarely can occur in MCTD.^2^ It commonly presents with accelerated hypertension, signs of hypertensive emergency, microangiopathic hemolytic anemia (MAHA), and rapidly progressive oliguric renal failure. Poor outcomes can be predicted with skin scores ≥20, diffuse cutaneous scleroderma (dcSSc), cardiac involvement, and patients who present in renal crisis with normotensive blood pressures.^3^ The current standard of care recommends aggressive blood pressure control with an angiotensin esterase inhibitor (ACEi) as the first-line agent in SRC as it has shown a >60% reduction in mortality.^3^ Angiotensin receptor blockers have not been proven as effective as ACEi. Furthermore, no proven benefit is seen with using ACEi and angiotensin receptor blockers prophylactically and its use is usually associated with poorer renal outcomes.^3^

We present a rare case of a patient with MCTD complicated by SRC with multiple poor prognostic indicators (normotensive, cardiac involvement, dcSSc, and skin score >20). We expanded our treatment to address each clinical sign independently, as the patient’s condition was deteriorating despite adhering to the standard of care, and our patient made a rapid renal recovery back to her normal baseline within 3 months.

## Case Report

A 50-year-old Filipino woman with a past medical history of MCTD, diagnosed 7 years prior, presented to the emergency department with complaints of shortness of breath, chest pain with a “bubble” like sensation, and diaphoresis with minimal exertion. She also had positive Raynaud’s phenomenon. Ten days prior she went to the emergency department for self-limited shortness of breath at which time her creatinine (CRE) was 0.8 mg/dL. Two months prior she was started on hydroxychloroquine 400 mg/day and prednisone 60 mg/day for a MCTD flare that manifested as muscle weakness. Her other home medications were amlodipine 5 mg/day, aspirin 81 mg/day, topical nitroglycerin to fingers 6 times/day as needed, alendronate 70 mg/week, and calcium carbonate/vitamin D_3_ daily. She has never smoked, never used illicit drugs, and denies consumption of alcoholic beverages.

The patient’s vital signs were unremarkable with a blood pressure reading of 116/80 mm Hg. Physical examination was remarkable for mild distress, a systolic murmur, crackles to mid lung fields, and a blanchable maculopapular rash on the chest.

Complete blood count reveled a leukocyte count of 9.1/mm^3^ and a platelet count of 205 000/mm^3^. Hemoglobin was 8.3 g/dL. Biochemical investigations were remarkable for elevated serum urea (53 mg/dL) and CRE (4.3 mg/dL). Liver enzyme tests revealed a total bilirubin of 1.5 mg/dL, an alanine transaminase level of 70 IU/L, and an alkaline phosphatase level of 124 IU/L. Serum lactate dehydrogenase was 343 U/L, and serum haptoglobin was 38 mg/dL (ref 30-200 mg/dL). Urine analysis revealed ++protein, ++white blood cells, 10 to 20 RBC/hpf (red blood cells per high-power filed), and granular casts. Urine microscopy was not done due to suspected diagnosis being MCTD flare. Her spot urine protein to creatinine ratio was 0.8 g/day. Her autoimmune workup was positive for speckled antinuclear antibody (1 in 2560), anti-RNP-topoisomerase I and -Smith antibodies. Complements C3 and C4 were decreased (39 and 7 mg/dL, respectively). Anti–double stranded DNA, -Scl 70, -JO1, -SS-A, -SS-B, and -centromere antibodies were negative. Cytoplasmic-staining (C)-ANCA and perinuclear-staining (P)-ANCA were both negative.

Abdominal ultrasound revealed gallbladder wall thickening. Computed tomography of the chest without contrast showed fibrotic inflammatory changes in the posterior basal segments, bronchial thickening with infiltrates suggestive of bronchopneumonia, an enlarged pulmonary artery, and moderate sized pleural effusions (right greater than left). Echocardiography showed an ejection fraction of 55%, a pulmonary artery pressure of 26 mm Hg, and a moderate sized pericardial effusion.

**Figure 1.**
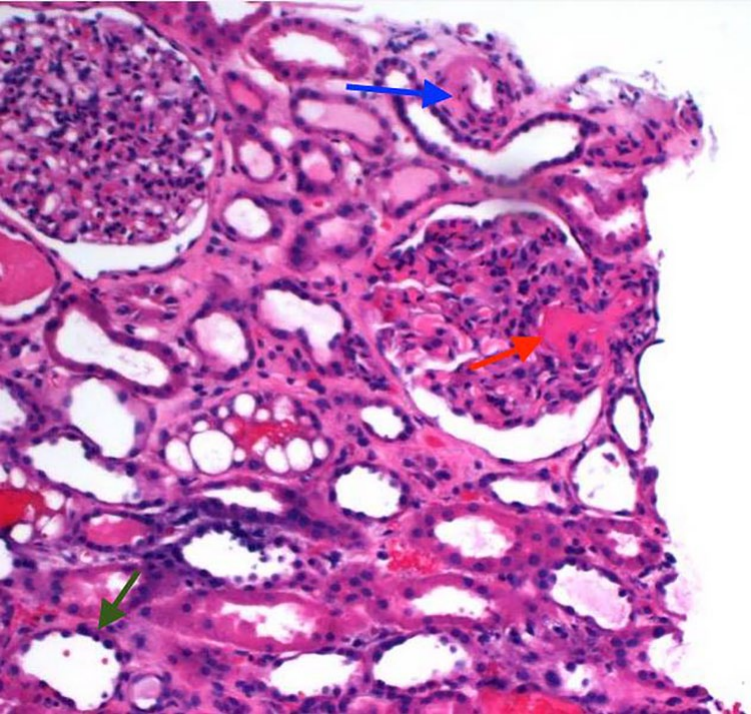
Light microscopy of kidney section stained with hematoxylin-eosin. The glomeruli display rare thrombi within capillary lumens and one has a thrombus within an afferent arteriole at the hilum (red arrow). A hyperplastic small vessel with narrowing of the tubular lumen is present (blue arrow). There is evidence of acute tubular injury with the dilation and flattening of the tubular epithelial cells (green arrow).

The patient was diagnosed with a MCTD flare, renal failure, and pneumonia. Hydroxychloroquine was discontinued. She was started on nifedipine, for worsening Raynaud’s phenomenon, methylprednisone 500 mg intravenous daily, vancomycin, and pipercillin/tazobactram. Her CRE continued to deteriorate (5.2 mg/dL), and her blood pressure continued to rise to 150/90 mm Hg. She was sent for a kidney biopsy. On day 4 methylprednisone was discontinued (for concern of precipitating SRC) and captopril (titrated to maximum dose within 72 hours) was started. On day 6 the patient developed oliguria and signs of hypervolemia. Hemodialysis was initiated every other day for a total of 6 sessions. The renal biopsy showed 9 available glomeruli with rare intraglomerular thrombi, moderately thickened vessels without onion skinning proliferation, or mucoid changes in their walls. No hypercellularity, necrosis, or crescent formation was seen. None of the glomeruli were globally sclerotic. One glomerulus had a thrombus within an afferent arteriole at the hilum (Figure 1). Multiple red cell casts within tubular lumina were noted with mild interstitial fibrosis. The tubules were dilated with flattening of the tubular epithelial cells, suggestive of marked acute tubular injury. Immunofluorescence microscopy revealed no deposition of IgG, IgA, IgM, C3, and C1q. There was no evidence of SRC or other forms of hemolytic-uremic syndrome (HUS)-type thrombotic microangiopathy. The findings of intraglomeruli thrombi were most compatible with thrombotic thrombocytopenia (TTP).

On day 8 the patient became acutely altered. Her complete blood count reveled a platelet count of 80/mm^3^ and hemoglobin level of 5.8 g/dL. Laboratory data revealed an elevated lactic acid dehydrogenase level of 466 U/L and a decreased haptoglobin level of <10 mg/dL. Complements C3 and C4 were both still decreased (42 and 6 mg/dL, respectively), and serum cardiolipin IgG was positive. A peripheral smear revealed fragmented red blood cells and anisocytosis. She was transfused multiple units of packed red blood cells without improvement. Due to the worsening MAHA along with the aforementioned presentation she was started on plasma exchange (using thawed plasma at 1.0 volume exchanges) for presumed thrombotic thrombocytopenia. On day 9 laboratory values for ADAMT-13 and atypical HUS (aHUS) were drawn and sent out. Methylprednisone 500 mg intravenous daily and mycophenolate at 1000 mg twice daily (chosen over cyclophosphamide due to lack of crescents, pauci-immune glomerulonephritis, and necrotizing inflammation on renal biopsy) were added to further control the MCTD flare. Treatment with eculizumab was considered but the patient refused. Daily pulse dose steroids at 500 mg was restarted with plasma exchange and continued (total of 6 exchanges received) until day 11. There was minimal response of a rise in platelets to plasma exchange with a count of 79/mm^3^ after her last treatment. Her last hemodialysis received was on day 13 at which time her CRE was 4.8 mg/dL. Over the month of hospitalization her serum CRE and urine output continued to improve and she was discharged with a CRE of 1.7 mg/dL and platelet count of 273/mm^3^. Her discharge medications were mycophenolate 500 mg twice daily, nifedipine extended release 60 mg/day, and lisinopril 20 mg/day. She was seen in clinic 6 weeks later, and her serum CRE had returned to 0.8 mg/dL. ADAMTS-13 activity later resulted as 67%, but was drawn after first plasma exchange session. The complement serologies for aHUS were sent to an outside speciality lab for processing, but the blood work was not resulted to improper collection.

## Discussion

MCTD with features of SRC is not commonly reported. From the previous 7 case reports worldwide on MCTD and SRC gathered, 3/7 cases became hemodialysis dependent and 4/7 cases responded to ACEi therapy (Table 1). A rare case of SRC in MCTD with pulmonary involvement was reported by Celikbilek et al,^4^ which differed from the previous 5 cases, in that this patient responded to a multidrug approach with enalapril, steroids, and immunosuppression medications versus enalapril alone. Our patient was rapidly declining despite being on an ACEi and high-dose corticosteroids.

**Table 1.** Summary of Previous Case Reports of MCTD and SRC and Their Outcomes^4,13,14-18^.

Case Report	Age (Years)	Sex	Presenting Symptom	Pathology Findings	Treatment	Outcome
Our case	54	Female	Chest pain, Raynaud’s phenomenon, AKI (CRE 4.6 mg/dL)	3/7 Intra-glomerular thrombi. Tubulo-interstitum with multiple red cell casts within tubular lumina. Mild interstitial fibrosis. Several moderately thickened vessels. No evidence of SRC or HUS type TMA, findings of intraglomeruli thrombi compatible with TTP	Plasma exchange, enalapril, HD, MMF, steroids	Responded to therapy
Vij et al^13^ (2014)	21	Male	Oliguria, SSc facies	Bloodless glomeruli, thickened glomerular capillary walls, interlobular vessels fibro-intimal hyperplasia with obliteration of capillary lumen, tubular injury and interstitial edema. Findings suggestive of SRC.	Plasma exchange and HD	HD dependent
Khan et al^18^ (2014)	36	Female	Blurry vision, arthralgias, and oliguric renal failure	14 glomeruli were seen which showed nonimmune complex–mediated disease process, ischemic collapse with fibrinoid necrosis suggestive of glomerular ischemic changes. Tubules reveled patchy degeneration with interstitial edema and hyaline casts.	Captopril	HD dependent
Khalil et at^14^ (2012)	44	Male	Hypertensive emergency, dyspnea, AKI (CRE 1.8 mg/dL)	2/11 sclerosed glomeruli, mild to severe capillary collapse in all other glomeruli. Intimal thickness in wall of blood vessels. The constellation of these findings wee suggestive of vasculopathy. IF revealed no deposition of IgG, IgA, IgM, C3 or C1q.	HD	HD dependant
Celikbilek et al^4^ (2007)	30	Female	Acute renal failure, pulmonary symptoms following abortion (CRE 4.1 mg/dL)	7/12 glomeruli globally sclerotic. Interstitial fibrosis and dense mononuclear inflammatory cell infiltration. Tubular atrophy. Arterial walls with prominent thickening and hyalinization. Findings compatible with SRC.	Enalapril, steroids, cyclophosphamide	Responded to therapy
Andersen and Vasko^15^ (2002)	64 (case 1); 45 (case 2)	Female (case 1); male (case 2)	2 cases both with features Raynaud’s phenomenon and pulmonary hypertension. Case 2 with right heart failure (CRE 2.9, 1.13 mg/dL, respectively).	Patient 2 kidney biopsy at autopsy showed renal interlobular arteries and arterioles with edematous, concentric, myxoid intimal proliferation and thickening. In a few vessels, almost total luminal obliteration. These findings were in accordance with SRC.	Enalapril	Both responded to therapy. Second case died from circulatory collapse.
Greenberg et al^16^ (2001)	64	Female	Inflammatory myopathy who developed SRC post steroid therapy	Active and severe TMA with extensive mesangiolysis and glomerular capillary wall remodeling with double contours in may glomeruli. Severe arterial and arteriolar sclerosis fibrin thrombi occlusion. Findings suggestive of a TMA.	HD	HD
Satoh et al^17^ (1994)	47	Female	Inflammatory myopathy with Raynaud’s phenomenon who developed acute kidney (CRE 1.2 mg/dL) injury with SRC	22 glomeruli with mild ischemic changes, no deposits, segmental lesions or crescents. Prominent vascular changes in 2 small arteries, 1/2 with complete occlusion by thrombi and the other with mild intimal proliferation. IF showed faint staining of IgM in the glomerular mesangium. These findings were suggestive of MCTD with SRC.	ACEi and prostaglandin inhibitors	Responded to therapy

Abbreviations: MCTD, mixed connective tissue disease; SRC, scleroderma renal crisis; AKI, acute kidney injury, CRE, creatinine; HUS, hemolytic-uremic syndrome; TMA, thrombotic microangiopathy; TTP, thrombotic thrombocytopenic purpura; HD, hemodialysis; MMF, mycophenolate mofetil; SSc, scleroderma; ACEi, angiotensin esterase inhibitor; IF, immunofluorescence; IgM, immunoglobulin M.

SRC can be precipitated by anemia, high blood pressure, cardiac events, cyclosporine initiation, or withdrawal, as well as high-dose steroids (greater than 15 mg/kg/day), which is thought to be due to the inhibition of prostacyclin production, which increases angiotensin converting enzyme activity, thus contributing to the development of SRC. Our patient had received high-dose steroids but worsening renal function was observed prior to use.

The overall biochemical picture in a patient with SRC is that of a thrombotic microangiopathic process. Laboratory findings in patients with SRC that may be observed include elevated plasma CRE (96%), MAHA (60%), and thrombocytopenia (50%).^5^ Urinalysis usually shows hematuria, proteinuria, and granular casts on microscopy.

Renal biopsies are not indicated in SRC, but are usually done to exclude other etiologies of renal failure when doubt exists.

In SRC, similar to malignant hypertension, primary small vessel thrombi (65%) usually predominate over glomerular thrombi (18%), in contrast to HUS and TTP.^5^ As the disease course varies the histological manifestations vary accordingly. Intimal accumulations of myxoid material, thrombosis, and/or fibrinoid necrosis are signs of early vascular changes.^5^ Onion-skin like lesions, which result from fibrointimal sclerosis with adventitial fibrosis, develop later in the course of SRC.^5^ When the disease reaches chronic stages, glomerular changes can vary from double contour and tram tracking to ischemic glomerular collapse.^5^ Although chronic changes of SRC and systemic sclerosis without SRC are difficult to differentiate on biopsy, patients with systemic sclerosis did not develop end-stage renal disease, which was reported over a mean follow-up of 10 years by Steen et al.^6^ This suggests that patients with SSc should be carefully evaluated for non-SSc causes of kidney disease. Approximately 61% of SRC patients have good outcomes when hypertension is aggressively controlled with ACE inhibitors.^7^

There are diagnostic difficulties differentiating among the other causes of thrombotic microangiopathy in MCTD. Such as in the case of our patient, TTP and aHUS became part of our differential diagnoses due to the lack of response to ACEi, worsening MAHA, change in mental status, and rapid decline in renal function. The high clinical suspicion for TTP or aHUS warrants early initiation with plasma exchange, which is life saving, without the luxury of an ADAMT-13 level or specific complement assays, respectively. It is of interest to note, although the findings of a normal ADAMTS-13 does not support the diagnosis of TTP, rare cases have been reported of MCTD complicated by TTP with normal ADAMTS-13 levels.^8^ Whereas plasma exchange is contraindicated with pure SRC, due to the possibility of a bradykinin mediated reaction resulting in severe hypotension, which is treated with the initiation of ACEi and strict blood pressure control.

It is worth mentioning that there is increasing evidence for the role of antibody-mediated injury in SSc/SRC. For one, particular clinical features have been found to be associated with disease-specific serum autoantibodies.^9^ Second, a select group of patients with SRC have been found to have anti-endothelial antibodies and increased expression of endothelin-1/endothelin-B^10^; endothelin overexpression has been discovered in antibody-mediated rejection in allograft kidneys.^11^ Last, several SRC kidney samples were found to have antiglobulin antibodies.^12^

## Conclusion

Normotensive SRC is a rare manifestation of MCTD with a poor outcome. The patient in our case worsened with conventional therapy for SRC and MCTD. Plasma exchange was initiated for a suspicion of superimposed TTP due to lack of response to treatment, worsening MAHA with thrombocytopenia, and change in mental status. Mycophenolate was initiated in addition to corticosteroids to control the underlying autoimmune disease. In light of all the poor prognostic indicators our patient made a full renal recovery in 3 months. Consideration should be given to controlling MCTD more aggressively, and the possibility that plasma exchange may have played a role.

## References

[1] Sharp GC Irvin WS Tan EM Gould RG Holman HR Mixed connective tissue disease—an apparently distinct rheumatic disease syndrome associated with a specific antibody to an extractable nuclear antigen (ENA). Am J Med. 1972;52:148-159. 4621694 10.1016/0002-9343(72)90064-2

[2] Denton CP Lapadula G Mouthon L Müller-Ladner U Renal complications and scleroderma renal crisis. Rheumatology (Oxford). 2009;48(suppl 3):iii32-iii35. doi:10.1093/rheumatology/ken483. 19487221

[3] Teixeira L Mouthon L Mahr A; Group Français de Recherche sur le Sclérodermie (GFRS). Mortality and risk factors of scleroderma renal crisis: a French retrospective study of 50 patients. Ann Rheum Dis. 2008;67:110-116. 17557890 10.1136/ard.2006.066985

[4] Celikbilek M Elsurer R Afsar B Ozdemir HB Sezer S Ozdemir NF Mixed connective tissue disease: a case with scleroderma renal crisis following abortion. Clin Rheumatol. 2007;26:1545-1547. 17119864 10.1007/s10067-006-0442-8

[5] Batal I Domsic RT Medsger TA Bastacky S Scleroderma renal crisis: a pathology perspective. Int J Rheumatol. 2010;2010:543704. doi:10.1155/2010/543704. 20981312 PMC2958499

[6] Steen VD Syzd A Johnson JP Kidney disease other than renal crisis in patients with diffuse scleroderma. J Rheumatol. 2005;32:649-655. 15801020

[7] Steen VD Medsger TA Jr. Long-term outcomes of scleroderma renal crisis. Ann Intern Med. 2000;133:600-603. doi:10.7326/0003-4819-133-8-200010170-00010. 11033587

[8] Suzuki E Kanno T Asano T Two cases of mixed connective tissue disease complicated with thrombotic thrombocytopenic purpura. Fukushima J Med Sci. 2013;59:49-55. 23842515 10.5387/fms.59.49

[9] Steen VD Autoantibodies in systemic sclerosis. Semin Arthritis Rheum. 2005;35:35-42. 16084222 10.1016/j.semarthrit.2005.03.005

[10] Kobayashi H Nishimaki T Kaise S Immunohistological study of endothelin-1 and endothelin-A and B receptors in two patients with scleroderma renal crisis. Clin Rheumatol. 1999;18:425-427. 10524561 10.1007/s100670050132

[11] Sis B Halloran PF Endothelial transcripts uncover a previously unknown phenotype: C4d-negative antibody-mediated rejection. Curr Opin Organ Transplant. 2010;15:42-48. 20009933 10.1097/MOT.0b013e3283352a50

[12] McCoy RC Tisher CC Pepe PF Cleveland LA The kidney in progressive systemic sclerosis: immunohistochemical and antibody elution studies. Lab Invest. 1976;35:124-131. 785092

[13] Vij M Agrawal V Jain M Scleroderma renal crisis in a case of mixed connective tissue disease. Saudi J Kidney Dis Transplant. 2014;25:844-848. 10.4103/1319-2442.135177 24969199

[14] Khalil AMM Iftikhar N Hussain SA Tan J Scleroderma renal crisis in a newly diagnosed mixed connective tissue disease resulting in a dialysis-dependent chronic kidney disease despite angiotensin-converting enzyme inhibition. CEN Case Rep. 2013;2:41-45. doi:10.1007/s13730-012-0036-z. 28509217 PMC5413725

[15] Andersen GN Vasko J Scleroderma renal crisis and concurrent isolated pulmonary hypertension in mixed connective tissue disease and overlap syndrome: report of two cases. Clin Rheumatol. 2002;21:164-169. 12086169 10.1007/s10067-002-8276-5

[16] Greenberg SA Amato AA Inflammatory myopathy associated with mixed connective tissue disease and scleroderma renal crisis. Muscle Nerve. 2001;24:1562-1566. 11745962 10.1002/mus.1184

[17] Satoh K Imai H Yasuda T Wakui H Miura AB Nakamoto Y Scleroderma renal crisis in a patient with mixed connective tissue disease. Am J Kidney Dis. 1994;24:215-218. 8048428 10.1016/s0272-6386(12)80185-5

[18] Khan I Khan I Ahmad T Noor H Mixed connective tissue disorder associated with scleroderma renal crisis. J Nephrol Ther. 2014;4:2.

